# Myelofibrosis-type megakaryocyte dysplasia (MTMD) as a distinct category of BCR::ABL-negative myeloproliferative neoplasms. Challenges and perspectives

**DOI:** 10.1038/s41375-023-01861-9

**Published:** 2023-03-04

**Authors:** Giovanni Barosi, Vittorio Rosti, Robert Peter Gale

**Affiliations:** 1 Center for the Study of Myelofibrosis, General Medicine 2, Istituto di Ricovero e Cura a Carattere Scientifico Policlinico S. Matteo Foundation, Pavia, Italy; 2 Centre for Haematology Research, Department of Immunology and Inflammation, Imperial College London, London, UK

**Keywords:** Haematological cancer, Haematopoietic stem cells

## Abstract

In this *Perspective*, we discuss criteria for defining a new disease entity or variant of a recognized disease or disorder. We do so in the context of the current topography of the BCR::ABL-negative myeloproliferative neoplasms (MPNs) where two new variants are reported: *clonal megakaryocyte dysplasia with normal blood values* (CMD-NBV) and *clonal megakaryocyte dysplasia with isolated thrombocytosis* (CMD-IT). The cardinal feature of these variants is bone marrow megakaryocyte hyperplasia and atypia corresponding the WHO histological criteria for primary myelofibrosis (*myelofibrosis-type megakaryocyte dysplasia-MTMD*). Persons with these new variants have a different disease course and features from others in the MPN domain. In a broader context we suggest myelofibrosis-type megakaryocyte dysplasia defines a spectrum of related MPN variants including CMD-NBV, CMD-IT, pre-fibrotic myelofibrosis and overt myelofibrosis, which differ from polycythemia vera and essential thrombocythemia. Our proposal needs external validation and we stress the need for a consensus definition of the megakaryocyte dysplasia which is the hallmark of these disorders.

We recently described two new disease variants we claimed belonging to the BCR::ABL-negative myeloproliferative neoplasms (MPNs) domain [1, 2]. The first is a re-formatting of our 1991 article reporting 18 cases of an a*typical myeloproliferative disorder with high risk of thrombosis and slow disease progression* [3]. The second is defined by a platelet concentration ≥450 × 10E + 9/L without other blood or laboratory signs of myeloproliferation or disease activity. The cardinal feature of both variants is bone marrow megakaryocyte hyperplasia and atypia corresponding the WHO description of megakaryocytes alterations included in the diagnostic criteria for primary myelofibrosis (PMF): *dense or loose clustering and frequent endosteal translocation of megakaryocytes with hyper-chromatic, hypo-lobulated, bulbous or irregularly folded nuclei and an aberrant nuclear/cytoplasmic ratio* [4]. (Fig. 1). We termed the first variant *clonal megakaryocyte dysplasia with normal blood values* (CMD-NBV) and the second *clonal megakaryocyte dysplasia with isolated thrombocytosis* (CMD-IT; Table 1).

**Fig. 1:**
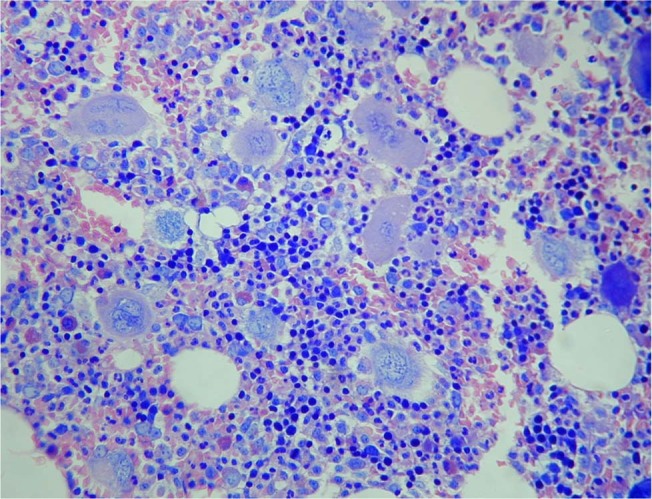
Bone marrow sample of a subject with clonal megakaryocyte dysplasia with normal blood values (CMD-NBV). The picture show megakaryocte hyperplasia and cellular atypia.

**Table 1:** The proposed diagnostic criteria of clonal megakaryocyte dysplasia with normal blood values (CMD-NBV) and of clonal megakaryocyte dysplasia with isolated thrombocytosis (CMD-IT).

**Clonal megakaryocyte dysplasia with normal blood values (CMD-NBV)**
*Subjects should be included in this category when all these criteria are met:*
1. Bone marrow megakaryocyte hyperplasia and dysplasia as described for primary myelofibrosis (*myelofibrosis-type megakaryocyte dysplasia*);^a^
2. Failure to meet the clinical-hematological WHO criteria for polycythaemia vera, essential thrombocythaemia, or primary myelofibrosis. Accordingly, subjects should not have a blood platelet concentration >450 × 10E + 9/L, or hemoglobin >165 g/L in men and >160 g/L in women and should not have any of the minor diagnostic criteria of primary myelofibrosis, i.e., leuko-erythroblastosis, increased serum LDH level, anemia or palpable splenomegaly.
**Clonal megakaryocyte dysplasia with isolated thrombocytosis (CMD-IT)**
*Subjects should be included in this when all these criteria are met:*
1. Bone marrow megakaryocyte hyperplasia and dysplasia as described for primary myelofibrosis (*myelofibrosis-type megakaryocyte dysplasia*);^a^
2. Sustained platelet concentration ≥450 × 10E + 9/L;
3. No minor WHO criteria for pre-fibrotic MF (anemia, palpable splenomegaly, WBC >11 × 10E + 9/L or increased LDH level).

^a^This criterion included megakaryocyte endosteal dislocation, loose and dense clustering, nuclear dense lobulation, cloudy and bare denuded nuclei, and nuclear-cytoplasmic abnormalities consistent with maturation defects. Abnormalities in the other haematopoietic cell lineages do not enter among the diagnostic criteria.

Our belief is that these reports represent an advance in the field of MPNs by reinforcing the concept of MPNs as an array of phenotypes and by highlighting that the number of these phenotypes is higher than previously recognized. This speculation is contrasted by the unavoidable limitations of our papers. These included retrospective analyses of a single center observational database without external validation, and bone marrow histology classified by one pathologist rather than by consensus amongst pathologists. However, the strongest criticism to our articles was these variants had insufficiently consistent phenotypic uniqueness to be considered new entities.

In the last few decades genetic studies of the MPNs indicate 3 underlying driver mutations, *JAK2*^V617F^, *CALR* and *MPL*. Data from next generation sequencing (NGS) has uncovered additional molecular complexity altering how we classify the MPNs. The revised 2017 and 2022 World Health Organization (WHO) criteria reflected these advances [4, 5]. For example, *pre-fibrotic myelofibrosis* (pre-fibrotic MF) has been added as has *MPN, unclassifiable*.

Despite this progress, classification of MPNs is not biology-driven. Patho-physiological mechanisms distinguishing polycythemia vera (PV) from essential thrombocythemia (ET) and pre-fibrotic MF from ET are incompletely defined nor understood. Consequently, as in many areas of medicine, classification of the MPNs follows the *statute of the nominal law* which prescriptively assigns names to entities with empirical repeatability. A disease becomes an entity if its existence is plausible without needing to know its causality or biology.

We think criticism of the new variants we described is more philosophical than biological. The underlying question is: when can we claim the existence of a new disease or disease variant? Our position is that the only relevant question for the acceptance is utility. The utility of our proposed new variants is that people with the features we describe have an unique disease course and features, and can be removed from the intellectually unsatisfying MPN, unclassifiable grab bag.

None of the 15 subjects with CMD-NBV had signs of disease progression after a median follow-up of more than 8 years, Subjects with CMD-IT lived longer than those with pre-fibrotic MF and those with overt- MF (HR = 0.42 [95% Confidence interval (CI) 0.23, 0.75], *P* = 0.003; and HR = 0.13 [0.075, 0.23], *P* < 0.001). Subjects with CMD-NBV had a significantly higher incidence of post-diagnosis thrombosis compared with persons with ET or PV (3.9 events *per* 100 subject-years *versus* 1.7 and 2.7). In contrast, subjects with CMD-IT had a significantly lower risk of thrombosis compared with persons with ET or pre-fibrotic MF [1.03 (0.53, 1.79) events *per* 100 subject-years *versus* 3.09 and 2.09].

Distinguishing features of the new variants we propose are of considerable clinical import. Moreover, they reinforce the concept of the MPNs as a spectrum of disorders promoted by specific constitutive and genetic features. For example, subjects with CMD-IT are more often female, have a higher frequency of type-2/type-2-like *CALR* mutations and a lower *JAK2*^V617F^ allele burden compared with persons with PMF. They also have a lower frequency of genetic variants correlated with risk of developing PMF such as the 46/1 haplotype and *VEGFA* rs3025039 polymorphism [3].

CMD-NBV is a special disease variant characterized by lack of hematological abnormalities or marginally elevated blood values. Subjects are mostly diagnosed in the context of thromboses. 13 of our 15 subjects with CMD-NBV had a synchronous symptomatic thrombotic event including portal vein thrombosis, Budd-Chiari syndrome, peripheral arterial thrombosis, myocardial infarction, spleen infarction or an incidentally detected portal cavernoma at diagnosis.

CMD-NBV forces us to rethink the generalizability of diagnostic reasoning in MPNs. The WHO criteria for the classical MPNs starts with considering an abnormal hematological value. In contrast, in CMD-NBV the diagnosis is triggered by clinical features. Positive predictive value of MPN diagnosis depends on patient- and practice setting-related co-variates. This results in a high level of uncertainty in CMD-NBV diagnostic reasoning requiring ad hoc rules to guide physicians to move from a circumstantial diagnostic metric to a metric based on clinical features, histology and genetics, and suggests the real frequency of CMD-NBV may be much greater than thought.

A further advancement in the MPN field of the introduction of the new disease variants is that they share the histological megakaryocyte characteristics typical of PMF, and that megakaryocyte morphology deviation was a major criterion to diagnose them. To define these megakaryocyte characteristics we now propose to introduce the term *myelofibrosis-type megakaryocyte dysplasia* (MTMD) and to highlight its relevance we conceptualize that this morphology identifies a disease category among MPNs.

The theory of MTMD has been accepted in the 2022 WHO [5] and International Consensus Classification (ICC) [6] to distinguish pre-fibrotic MF from ET. With our proposal, now MTDT defines a group of related MPN variants including CMD-NBV, CMD-IT, pre-fibrotic MF and overt MF (Fig. 2). In our dataset these variants constitute 2, 13, 37 and 48 percent of this category. If representative, our data suggest the indolent non-fibrotic variants are more common than overt MF.

**Fig. 2:**
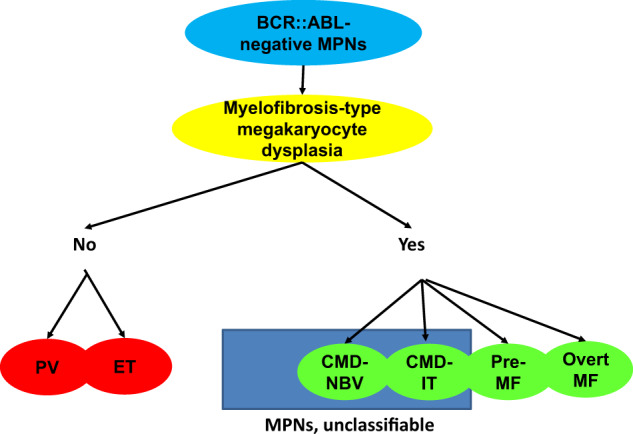
The proposed classification of BCR::ABL-negative myeloproliferative neoplasms. PV polycythemia vera, ET essential thrombocythemia, CMD-NBV clonal megakaryocyte dysplasia with normal blood values, CMD-IT clonal megakaryocyte dysplasia with isolated thrombocytosis, pre-MF pre-fibrotic myelofibrosis, overt MF overt myelofibrosis.

This MTMD paradigm, born from an acute morphological vision, is now supported by biological evidence. In fact, hypo-morphic *GATA1* mutations selectively decrease GATA1 in megakaryocytes and induce myelofibrosis in mice and a bone marrow histology like primary myelofibrosis in humans [7]. Also, megakaryocytes from humans with PMF have low levels of GATA1 probably maintained by ribosome abnormalities induced by driver mutations [8]. Recently, aurora kinase A was reported to be over-expressed in PMF and a selective inhibitor promotes polyploidization and differentiation of megakaryocytes with PMF-associated mutations in mice [9]. Finally, evidence that megakaryocyte morphology in PMF is unique among MPNs is suggested by the observation that in vitro PMF-derived megakaryocytes display nuclei with a bulbous appearance, and are smaller than those ET- or PV-derived [10].

The MTMD concept contrasts with megakaryocyte dysplasia in other myeloid disorders such as myelodysplastic syndromes (MDS). Histological criteria distinguishing megakaryocyte dysplasia in MDS from that in PMF is proposed but unvalidated [11]. A useful classification tool could be the megakaryocyte dysplasia score proposed to predict response in people with PMF receiving a haematopoietic cell transplant but is unvalidated in non-transplanted persons [12].

In conclusion, the recognition of 2 new variants of MPN including subjects previously considered under the rubric of *MPN, unclassifiable* addressed us to hypothesize a broad category of MPNs bearing different phenotypes may be included in an unique category according to the megakaryocyte morphology. Agreeing on MTMD as a category needs standardization of relevant bone marrow features. Since the histological assessment of bone marrow in MPNs remains constrained by a reliance on subjective and qualitative criteria, we emphasize the use of more precise methods. Computational methods designed to systematically capture the key morphological characteristics of megakaryocytes were proved able to associate with particular MPN subtypes [13, 14]. This strategy seems to have significant potential for translation into a better definition of our proposed category of megakaryocyte morphology. Biomarkers of this histological diagnostic criteria would also help and should be investigated.
